# Evaluation of a new body-focused group therapy versus a guided self-help group program for adults with psychogenic non-epileptic seizures (PNES): a pilot randomized controlled feasibility study

**DOI:** 10.1007/s00415-021-10652-0

**Published:** 2021-06-18

**Authors:** Philine Senf-Beckenbach, Matthias Hoheisel, Janine Devine, Arnina Frank, Laura Obermann, Matthias Rose, Kim Hinkelmann

**Affiliations:** 1 Center of Internal Medicine and Dermatology, Department of Psychosomatic Medicine, Charité-Universitätsmedizin Berlin, corporate member of Freie Universität Berlin, Humboldt-Universität zu Berlin, and Berlin Institute of Health, Charitéplatz 1 (Sauerbruchweg 5, 2. Ebene), Campus Charité Mitte, 10117 Berlin, Germany; 2 AOK Institute, Berlin, Germany

**Keywords:** Psychogenic non-epileptic seizures, Dissociative seizures, Body psychotherapy, Group psychotherapy treatment, Randomized clinical feasibility study

## Abstract

**Objective:**

Psychogenic non-epileptic seizures (PNES), a common phenomenon in neurological settings, are regarded as a paroxysmal type of functional neurological disorder (FND). In a substantial proportion, PNES are disabling with poor long-term outcomes and high economic costs. Despite the clinical and financial consequences of PNES, there is still a lack of controlled clinical trials on the treatment of this challenging disorder. The study aims to evaluate the feasibility and collect first evidence of the efficacy of a group based-intervention in PNES-patients.

**Methods:**

A pilot randomized controlled feasibility study with a parallel-group design was performed in adult outpatients with PNES to evaluate a new body-focused group therapy (CORDIS) versus guided self-help groups. Self-assessment of dissociation (Dissociation Experience Scale—DES-20) and seizure severity (Liverpool Seizure Severity Scale—LSSS) were assessed two weeks before and two weeks after the treatment intervention and also six months after treatment as primary outcome parameters.

**Results:**

A total of 53 patients were recruited from a specialized outpatient clinic, and out of those, 29 patients completed either the body-focused group therapy program (*n* = 15) or a guided self-help group (SHG) therapy (*n* = 14). When analyzing the ITT sample (*n* = 22 CORDIS group, *n* = 20 SHG), both groups showed an effect on seizure severity and level of dissociation. In the per protocol sample (*n* = 13 CORDIS group, *n* = 12 SHG), CORDIS was superior to the self-help group for reducing seizure severity 6 months after the treatment.

**Significance:**

CORDIS is a newly developed body-focused group therapy program for adults with PNES. Further studies should include a multicentric design with a higher number of participants.

## Introduction

Psychogenic non-epileptic seizures (PNES) are paroxysmal episodes characterized by the loss of voluntary control over body functions that are usually intentional. Non-epileptic seizures resemble epileptic seizures but are not related to abnormal electrical activity in the brain [1]. The prevalence of PNES in the general population has been estimated at 2–50/100.000 [2]. In general neurology outpatient clinics, PNES account for 2% of new referrals [2]. In tertiary Epilepsy units, the proportion of PNES among all patients is around 30% [3]. The aetiology of PNES is still subject to research. Theoretical psychological models assume that PNES occur in response to distressing stimuli when alternative coping mechanisms are inadequate or have been overwhelmed [1]. The occurrence of PNES is often associated with psychological trauma and posttraumatic stress disorder [4]. Current treatment recommendations rely on psychological interventions after a thorough workup. Antiepileptic drugs are neither indicated nor helpful in PNES [5]. Although caring for PNES patients thus usually demands an interdisciplinary approach, they are often solely treated either in neurological or in psychiatric/psychosomatic settings, leading to disadvantages in treatment quality.

Despite the high frequency of PNES in clinical settings, there are surprisingly few studies of high methodological quality focusing on guideline-oriented treatment options for PNES. Three previous pilot treatment trials have focused on cognitive behavioural therapy (CBT) programs for PNES patients [7–9]. The first two monocentric studies showed that a structured CBT treatment may reduce the seizure frequency in PNES [7, 8]. There is one multicentric study from 2020 involving 368 patients [9]. This study revealed that a structured CBT treatment plus standard medical care was superior to only standard medical care regarding the outcome of the burden of somatic symptoms, bothersomeness of seizures, the general quality of life, work and social life functioning, and overall psychological distress [9]. Based on those pilot trials in the English-speaking area, we developed a body-focused group therapy program in German. Its content is presented in detail in a previous publication [10], including cognitive-behavioural aspects that are already established in the existing English treatment programs [7–9]. A new aspect is that it also involves a body-focused psychotherapy approach with symptom-specific exercises. This body-focused approach refers to the German guidelines for the treatment of functional body symptoms. In those guidelines, the involvement of body-focused psychotherapy is recommended due to the body-focused symptomatology of the disease [6].

In this study, we performed a randomized controlled feasibility trial to evaluate the effects on seizure severity and dissociation level of our treatment program CORDIS for PNES patients. We compared these effects with those of a guided self-help group (SHG), an effective therapy option for psychological diseases with expected nonspecific treatment effects [10]. We hypothesized that our program is superior to an SHG in decreasing seizure severity and level of dissociation in patients with PNES.

## Methods

### Trial design

The primary objective was to estimate the effect of the manualized group treatment program 'CORDIS' on seizure frequency compared to that of SHG in a parallel-group study design. After the inclusion of the first three patients, we changed our primary outcome parameter from “seizure frequency” to “seizure severity” and the “level of dissociation”, based on the results of psychometric assessment tests. The reason for this change of study design were difficulties with assessing seizure frequencies include high variability (multiple seizures per day versus one seizure per month), lack of valid scales and instruments, and different definitions of when to count symptoms like seizures. This resulted in high discrepancies between the seizure documentation by patients and the actual interpretation of the seizure situation from the evaluating physician.

Further, we established an active control-group design based on the decision framework for randomized trials of behavioural interventions in psychiatry [11]. All participants provided informed consent for research participation. All data were collected and interpreted at Charité University Medicine Berlin. The study was approved by the Ethics Committee of Charité University Medicine Berlin (EA1/185/16). Registration as a randomized clinical trial was prospectively initialized in the German registry for clinical studies on 06/04/2018 (DRKS, study number DRKS00014251).

### Power calculation

For calculating the case number, we referred to the monocentric study by Goldstein et al. in which a comparable study design was used for an individual intervention [8]. We calculated Cohen's *F* [25], which resulted in an effect size of 0.36. On the basis of this effect size, a total sample number of *N* = 18 patients was calculated to identify a treatment difference between the two treatment arms with a probability of 80% and a two-sided significance level of 0.05. It has to be noted that the highly significant difference between the study groups in the 2010 study is likely to be partially due to a bias in time and attention since the intervention group with 10 therapy sessions received significantly more attention than the control group (treatment as usual). In our study plans, we attempted to avoid this bias by providing an SHG within the same time frame. Accordingly, we expected a lower effect than in the 2010 study and corrected the improvement in the seizure frequency in the control group from 1.25 to 7 per month. With these corrected raw data, a Cohen's *F* of 0.2 was calculated. On the basis of this assumed effect size, a total sample number of *N* = 52 patients was required to detect a mean difference between the two treatment arms with a probability of 80% and a two-sided significance level of 0.05. With an expected dropout rate of 15%, 60 patients had to be recruited (30 patients per group).

### Participants

We recruited patients from a specialized outpatient clinic for PNES patients at Charité University Berlin, Department of Psychosomatic Medicine. Adults (> 18 years) that met the following inclusion criteria: PNES diagnosed by an experienced epileptologist via 24-h continous video-electroencephalography, seizure videos and/or description of the seizures from relatives as well as ongoing seizures for more than six months with at least one seizure every 2 months and at least one seizure 4 weeks prior to the start of the intervention. Exclusion criteria were comorbidity of epilepsy, current psychotherapeutic treatment, psychosis, substance abuse, acute suicidality, insufficient language skills, and inability to complete the questionnaires (e.g. blindness).

### Randomization and dropout

We performed computer-generated block randomization using the program Randomizer (www.randomizer.org). Randomization and enrollment of patients were performed by different members of our study group. All participants received detailed information about the study and written consent was obtained.

### Demographic and clinical characteristics

All participants completed questionnaires to capture demographic data, psychiatric and somatic pre-existing conditions, age, gender and level of education. In addition, the Childhood Trauma Questionnaire (CTQ) was used to determine early-life traumatization. The CTQ is one of the most commonly-used and well-validated measures for early-life traumatic events [22].

### Outcome parameters

We measured potential treatment effects at baseline two weeks before the treatment program started (pre-treatment), 14 days after treatment (post-treatment) and six months and two weeks after treatment (follow-up). We defined the level of dissociation and seizure severity as primary outcome parameters. Secondary outcome parameters were depressive symptoms and somatoform symptoms [17].

#### Primary outcome parameters

##### Level of dissociation

We measured the level of dissociation with the Dissociation Experience Scale—20 (DES-20). The DES-20 (German version: FDS-20) is a 20-item scale rated in percentage from 0 to 100, which measures the frequency of dissociative experiences [13]. The total DES-20 score is the mean of all item responses. The scale considers a wide range of dissociative symptoms but focuses on those regarding awareness and consciousness. The German version showed good internal consistency [14].

##### Seizure severity

We measured seizure severity with the Liverpool Seizure Severity Scale (LSSS). The LSSS is a 20-item self-report questionnaire to assess the severity of seizures within the past four weeks. As a supplement question to the LSSS, we added a question about the seizure frequency in the past year. The LSSS is scored from 0 to 100, with higher scores reflecting greater seizure severity. The LSSS showed good internal consistency (*α* = 0.72–0.96) in patients with epilepsy [15] and has been used in patients with PNES [16].

#### Secondary outcome parameters

##### Depressive symptoms

We measured depressive symptoms with the the Patient Health Questionnaire (PHQ-9). The PHQ-9 is a self-report instrument and screens for depressive symptoms. It consists of 9 items with a total summation score of 27. Spitzer et al. published the reliability and validity studies [18].

##### Somatoform symptoms

Somatoforms symptoms were measured with the Patient Health Questionnaire for somatoform symptoms (PHQ-15). It consists of 15 items, with a total summation score of 30. The PHQ-15 has been shown to have good internal consistency (*α* = 0.8) [19].

## Interventions

### Body-focused group therapy program CORDIS

The manualized group treatment program CORDIS was developed to fill the gap of specific treatment options for PNES patients in Germany. The name “CORDIS” stands for CORpus (referring to bodyfocused therapy) and DISsociation. It was developed by three authors with expertise in epileptology, neuropsychosomatics and psychotherapy and is partly based on existing programs for English natives [7, 8]. It consists of a combination of measures for psychoeducation, emotion-regulation and body perception. CORDIS consists of 10 weekly sessions of 90 min each. Each session is structured with repetitive elements such as greeting rituals, discussion of the symptom diaries and guided performance of the group exercises. Each session contains a detailed plan for every therapy session, see Table 1. In our study, the treatment was performed by two experienced group therapists who received training in how to use the CORDIS manual. Adherence to the manualized intervention strategy was supported through regular supervision provided by the authors of the manual.

**Table 1:** CORDIS treatment program

Session	Content to be discussed	Body psychotherapy and exercises
Session 1	Salutation and welcome round Explanation of symptom diary Compliance as a main factor for success of program	Grounding exercise
Session 2	Symptom diary What is dissociation? Getting to know disease models (1)	Grounding exercise Painting the feeling of the seizure on a pre-printed sheet (body sketch)
Session 3	Symptom diary Getting to know disease models (2) Creating an individualized disease model for every patient	Grounding exercise
Ssession 4	Symptom diary When does my seizure occur? (1) Tension curve (Linehan) Concept of triggers (1)	Grounding exercise Exercise to explore inner state of tension
Session 5	Symptom diary When does my seizure occur? (2) Early signs of a seizure Concept of trigger (2)	Grounding exercise Exercise to explore individual external borders (to other people)
Session 6	Symptom diary Concept of skills (1)	Grounding exercise Exercise with potential triggering effect to explore the beginning of a seizure (and practise skills)
Session 7	Symptom diary Concept of skills (2) Packing the individual skills suitcase for every proband	Grounding exercise Exercise to explore emotional states as potential triggers
Session 8	Symptom diary Topic emotion: emotion recognition (potential triggers), naming and regulation of emotions	Grounding exercise Exercise to explore negative thoughts as potential triggers
Session 9	Symptom diary Topic negative thoughts: automatic negative thoughts (as potential triggers)	Grounding exercise Group decision on the repetition of previously exercises
Session 10	Symptom diary Saying good bye Motivation for following longterm psychotherapy	Grounding exercise Group decision on the repetition of previously exercises

Overview of therapy sessions from the CORDIS treatment program

### Self-help group (control intervention)

The guided SHG consisted of 10 weekly sessions of 90 min each. Each session was introduced by a trained student. In the first group session, the student led a round of introductions. Then, the student introduced a general topic with advice on how to discuss the issue (e.g. mind mapping). The topics of discussion provided by the students were as follows: anxiety of seizures, feelings of embarrassment regarding seizures, loss of control, taking control over seizures, helpful proxies, seizures and the family, triggers for seizures, pessimism or optimism and finding helpful resources to deal with seizures. The last session consisted of a summary from the previous sessions and a farewell round without any specific topic.

### Publications

We reported interim results of our study only from the intervention group in an earlier publication [10]. Based on the patient collective of our study some other studies with a cross-sectional design were performed and published [28–30].

### Statistical analysis

We analyzed all data using SPSS, Version 24.0 (IBM Corp. Armonk, NY). Normality in distribution and potential outliers of all outcome scores were assessed with histograms. Demographic characteristics between intervention groups were compared using univariate analysis of variance (ANOVA) for continuous variables and *χ*^2^ tests for dichotomous variables. Missing data in our Intention-to-treat (ITT) sample were imputed with the last observation carried forward (LOCF) approach. Due to high dropout rate in the ITT sample we also conducted all analyses in the per protocol sample (patients completing pre and post-intervention assessments, *n* = 25). Follow-up data was calculated based on the per protocol sample due to extremely high dropout numbers. For main analyses, we calculated change scores of pre-treatment versus post-treatment (two weeks after completion of the treatment intervention). For these we also calculated Bias Corrected accelerated 95% Confidence Intervals (BCa 95% CI) based on 1000 bootstrap samples. We conducted separate ANCOVAS with change scores as outcome variables and intervention (Cordis vs SHG) as group variable, controlling for pre-treatment scores. We also calculated the number of responders (25% reduction of outcome score) in both intervention groups.

Follow-up assessment scores were compared with a repeated measures ANOVA with three time points (pre-treatment, post-treatment, follow-up) as within subject factor and intervention group as between-subject factor.

We also calculated effect sizes (partial *η*^2^) and their 90% confidence intervals (CI). We defined effect sizes according to Cohen (1988) as small (*η*^2^ = 0.01), medium (*η*^2^ = 0.06), and large (*η*^2^ = 0.14) effects [25, 26]. If not stated otherwise, the level of significance was set at *p* < 0.05.

## Results

### Participant flow

**Fig. 1:**
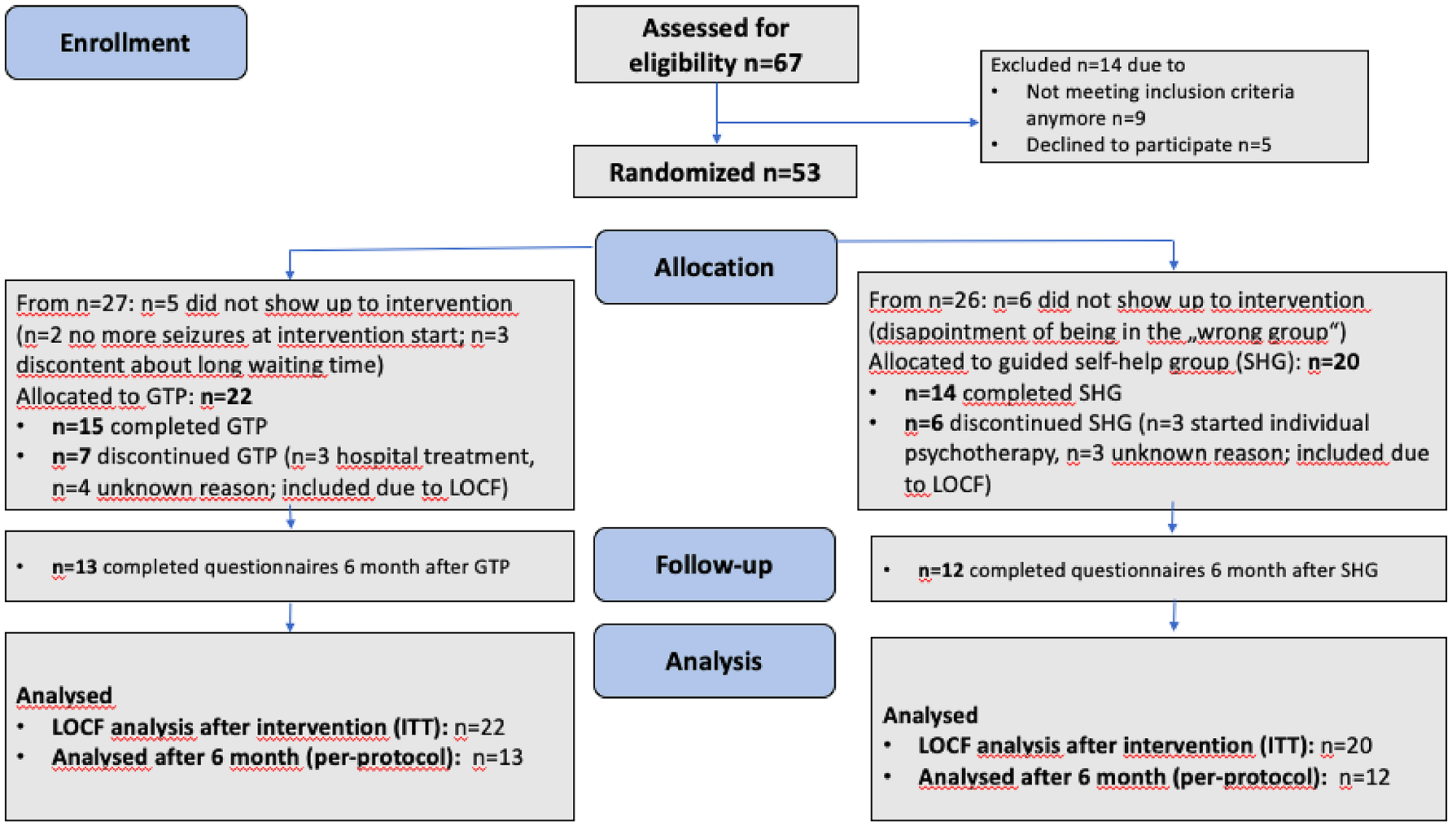
Consort flow diagram of the RCT study (*LOCF* last information carried forward, *GTP* group treatment program, *SHG* self-help group, *n* number)

For details of recruitment and dropout see also Fig. 1 (CONSORT flow diagram). Sixty-seven patients were screened for eligibility between May 2018 and December 2018. Five patients declined to participate for unknown reasons and nine patients did not meet the inclusion criteria (four patients started psychotherapy, the diagnosis of two patients revealed unclear seizure etiology, and three patients did not have any more seizures for more than three months). The remaining 53 patients were recruited and randomized 1:1 to one of the two treatment arms (*n* = 27, group treatment program (CORDIS); *n* = 26, SHG). After randomization, 11 patients withdrew their informed consent and refused to participate in further visits or follow-ups, thus pre-treatment assessments could not be obtained. Reasons for withdrawal were spontaneous symptom reduction (CORDIS group, *n* = 2), discontent with the treatment plan (long waiting time) (CORDIS group, *n* = 3) and disappointment of being put in the 'wrong' group (SHG, *n* = 6). Our ITT sample, therefore, consisted of all patients, that were randomized and provided pre-treatment data (*n* = 42) with 22 patients in the CORDIS group and 20 patients in the control group (SHG).

Thirteen patients interrupted the study; seven patients from the treatment group (three patients needed hospital treatment and the remaining four left for unknown reasons) and six patients from SHG (three patients started individual psychotherapy during the study and the remaining three left for unknown reasons). Twenty-nine participants completed either the treatment program (*n* = 15) or SHG (*n* = 14), but only 25 patients completed the questionnaires and could therefore be included in the per-protocol analysis (*n* = 13 CORDIS group, *n* = 12 SHG).

### Demographic and clinical characteristics

The CORDIS and control group did not differ significantly in terms of demographic and clinical characteristics (see Table 2). Regarding seizure frequency 14 days prior to the beginning of the intervention from all 42 patients, *n* = 24 patients stated to have seizures every day, *n* = 15 patients had 3–5 seizures per week, two patients had one seizure per week and one patient had only one seizure in the last two weeks before assessment.

**Table 2:** Sociodemographic and clinical characteristics

	CORDIS group (*n* = 22)	SHG group (*n* = 20)	*p* value
Age in yrs, mean (± SD)	36.6 (12.1)	32.8 (13.2)	0.08
Gender *n* (%)			0.06
Female	19 (86.4)	12 (60)	
Male	3 (13.6)	8 (40)	
Education in yrs, mean (± SD)	11.8 (1.6)	11.2 (1.6)	0.24
Duration of disease in yrs, mean (± SD)	6.5 (6.7)	10.7 (10.4)	0.15
Pack years (nicotine), mean (± SD)	1.1 (3.1)	6.0 (14.2)	0.15
Childhood Trauma Questionnaire Total Score, mean (± SD)	53.3 (15.5)	54.2 (23.7)	0.89

Clinical characteristics of patients at baseline (pre-treatment) in the intervention and the active control group

*p* value calculated with univariate analysis of variance (ANOVA) for continuous variables and *χ*^2^ tests for dichotomous variables

*SD* standard deviation, *yrs* years, *SHG* self-help group, *PHQ* Patient Health Questionnaire, *PD* personality disorder

### Primary outcome parameters

All primary and secondary outcome parameters were calculated based on the ITT sample (*n* = 22 CORDIS, *n* = 20 SHG) in consent to the “last information carried forward”—method. All outcome variables (means, SD) for all time points are shown in Table 3.

**Table 3:** Level of dissociation and seizure severity (primary outcome) at pre- and post-treatment and follow-up

	CORDIS group	SHG group	*p* value*
FDS-20 Scores, mean (SD)			
Pre-treatment	26.1 (16.3) (n = 22)	23.2 (16.8) (n = 20)	
Post-treatment	21.1 (12.8) (n = 22)	22.2 (15.7) (n = 20)	0.15
Follow-up (6 month and 14 days)	19.8 (10.9) (n = 13)	16.8 (10.8) (n = 12)	0.43
LSSS Scores, mean (SD)			
Pre intervention	48.14 (6.7) (n = 22)	51.6 (4.5) (n = 20)	
Post intervention	46.6 (7.4) (n = 22)	49.5 (7.0) (n = 20)	0.83
Follow-up (6 month and 14 days)	40.3 (8.0) (n = 13)	48.8 (6.6) (n = 12)	0.03

Comparison of pre- and post-treatment scores (ITT sample, LOCF imputation) and follow-up (per protocol sample)

*p* value for post-treatment calculated with ANCOVA for difference score and adjusted for pre-treatment FDS/LSSS-values; *p* value for follow-up calculated with repeated measures ANOVA with three timepoints

*SD* standard deviation, *SHG* self-help group, *n* number, *LSSS* Liverpool Seizure Severity Scale, *FDS* German version of Dissociation Experience Scale (Fragebogen dissoziativer Symptome)

### FDS-20

Comparing pre and post-intervention scores with respect to dissociation (FDS-20), patients in the CORDIS group improved with a mean of 4.98 (SD 8.73) points (BCa 95% CI [1.56, 9.01]), patients in the SHG improved with a mean of 0.93 (SD 7.61) points (BCa 95% CI [− 2.32, 4.84]). ANCOVA, adjusting for pre-intervention FDS-20 scores, revealed no significant effect of the intervention (*F* (1, 40) = 2.89, *p* = 0.15, partial *η*^2^ = 0.05, 90% CI [0.00, 0.20]), with effect size indicating a small to medium effect.

Comparing response rates (25% improvement) revealed an equal number of responders in the CORDIS group (*n* = 5) and the SHG group (*n* = 4) (*χ*^2^ (1) = 0.09, *p* = 1.00). Repeating the analyses in the per protocol sample yielded the same results with a mean FDS-20 change score of 8.04 (SD 10.00) points (BCa 95% CI [3.35, 13.89]) in the CORDIS group versus 1.42 (SD 9.53) points (BCa 95% CI [− 4.04, 6.44]) in the SHG (ANCOVA *F* (1, 26) = 1.53, *p* = 0.23, partial *η*^2^ = 0.06, 90% CI [0.00, 0.25]).

### LSSS

Comparing pre and post-intervention scores with respect to seizure severity (LSSS), patients in the CORDIS group improved with a mean of 1.59 (SD 4.10) points (BCa 95% CI [0.06, 3.13]), patients in the SHG improved with a mean of 2.20 (SD 5.67) points (BCa 95% CI [0.52, 4.22]). ANCOVA, adjusting for pre-intervention LSSS scores, revealed no significant effect of the intervention group (*F* (1, 41) = 0.05, *p* = 0.83, partial *η*^2^ = 0.001, 90% CI [0.00, 0.06]). The number of responders was equal (*n* = 1 in each intervention group) (*χ*^2^ (1) = 0.05, *p* = 1.00).

Repeating the analyses in the per protocol sample yielded the same results: Mean LSSS change score of 2.36 (SD 5.01) points (BCa 95% CI [− 0.08, 5.08]) in the CORDIS group versus 2.84 (SD 6.70) points (BCa 95% CI [0.29, 6.81]) in the SHG (ANCOVA *F* (1, 26) = 0.01, *p* = 0.91, partial *η*^2^ = 0.001, 90% CI [0.00, 0.02]).

### Secondary outcomes

Comparing pre and post-intervention scores with respect to depressive symptoms, PHQ-9 yielded no significant results (change scores PHQ-9 were 0.52 (SD 2.86) for CORDIS group vs. 1.50 (SD 3.49) for SHG; ANCOVA (*F *(1, 41) = 0.89, *p* = 0.35, partial *η*^2^ = 0.02, 90% CI [0.00, 0.14]).

Improvement of somatoform symptoms (PHQ-15) was significantly higher in SHG (mean 2.35 points, SD 4.24) compared to CORDIS group (mean 0.10 SD 3.28) (ANCOVA (*F *(1, 41) = 3.96, *p* = 0.05, partial η^2^ = 0.09, 90% CI [0.00, 0.24]).

No harms or unintended effects were observed, neither in the intervention nor in the control group.

### Follow-up

Follow-up assessments were compared in the per protocol sample only (*n* = 13 CORDIS, *n* = 12 SHG).

Repeated-measures ANOVA with FDS-20 as dependent variable revealed a significant effect of time (*F*(2;22) = 4.48, *p* = 0.02, partial *η*^2^ = 0.16, 90% CI [0.02, 0.31]), indicating an improvement from pre-treatment to follow-up across groups, but no effect of group (*F*(2;22) = 0.78, *p* = 0.39, partial *η*^2^ = 0.03, 90% CI [0.00, 0.13]) and no significant interaction effect (*F*(2;22) = 0.85, *p* = 0.43, partial *η*^2^ = 0.04, 90% CI [0.00, 0.13]).

For LSSS, repeated measures ANOVA revealed a significant effect of time (*F*(2;22) = 7.12, *p* = 0.002, partial *η*^2^ = 0.24, 90% CI [0.06, 0.38]) reflecting an improvement in LSSS scores across groups. The group effect was not significant (*F*(1;22) = 3.67, *p* = 0.07, partial *η*^2^ = 0.14, 90% CI [0.01, 0.28]). There was a significant time by group interaction (*F*(2;44) = 4.64, *p* = 0.03, partial *η*^2^ = 0.17, 90% CI [0.02, 0.31]) indicating greater improvement of LSSS scores in the CORDIS group (“group”) compared to SHG from pre-treatment to follow-up (“time”).

## Discussion

The present study is the first to evaluate a symptom-specific treatment program for German patients with PNES in a randomized clinical trial design. It features a new therapeutic aspect by including body-focused therapy exercises next to well-established cognitive-behavioural treatment methods. Furthermore, it is the first study that compares the effects of psychotherapeutic treatment for PNES with an active control treatment (guided SHG).

Patients who participated in the CORDIS treatment program and SHG showed improved dissociation levels and reduced seizure severity after the respective intervention (ITT sample analysis). However, there was no significant difference in the efficacy between either of the interventions, but the effect size showed a small to medium effect. After the six-month follow-up testing, the CORDIS treatment was superior to the active control intervention regarding the outcome parameter 'seizure severity' (per protocol analysis). Our study adds evidence to the currently small spectrum of treatment options for PNES patients and points toward the notion that body focussed interventions may be effective in PNES.

According to Röhricht et al., body psychotherapy is characterized by the central guiding principle that the body remains the focus of the therapeutic work. This counts especially for diseases with body-focused symptomatology, as given in PNES [12]. PNES patients might get irritated if therapists confront them directly with explanations concerning the psychological genesis of their disease. It can be confusing that the seizures in PNES are caused by psychological reasons because patients tend to experience them clearly as a body dysfunction. Therefore, CORDIS tries not to directly address psychological theoretical explanation models unless the patient brings them up [12]. This theoretical approach in combination with repeated exercises helping to improve body perception might play a role in the positive long-term effects of our treatment program, as shown in other studies focusing on body psychotherapy for medically unexplained symptoms [27].

In contrast to earlier studies we used seizure severity and not seizure frequency as outcome parameter. This may reduce comparability, however, there is growing evidence suggesting that seizure frequency alone may not be the most important determinant of quality of life in patients with PNES [9, 16].

Interestingly, the only available multicentric study on the efficacy of a treatment program for PNES patients showed that seizure frequency in PNES patients did not improve significantly in a CBT-treated group, compared with that in the standard medical care group, however somatic symptom burden in general did [9].

In a study from 2005, the usefulness of seizure frequency as an outcome parameter had been already questioned [24]. Difficulties with assessing seizure frequencies include high variability, lack of valid scales and instruments and different definitions of when to count symptoms as seizures resulting in discrepancies between the seizure documentation by patients in a diary and the actual interpretation of the seizure situation from the evaluating physician (epileptologist/study physician). Furthermore, a lot of patients experience strong derealisation or amnestic states making subjective ratings difficult to rely on. We, therefore, chose seizure severity, measured with a standardized instrument of epileptic seizures (LSSS) as the outcome parameter in our study. However, we are aware of the shortcomings of using a scale for epilepsy in patients with PNES.

Another difference to earlier studies is that we used an active control group. First, we believe that using an active control group is a strength of our study because it tests for the so-called nonspecific treatment factors. Several studies have shown that common (nonspecific) factors cause an average placebo response of around 40% in psychotherapy. This condition is comparable to drug RCT studies under similar conditions when primary outcome efficacy measures are patient-reported outcomes [18–20]. These nonspecific effects might play a role in the very high significant effects of previously evaluated treatment programs for PNES patients [7, 8].

Gold et al. describe a framework in which they offer an algorithm that helps construct clinical psychiatric studies. Considering this framework for our study condition, an active and nonspecifically effective control group, especially for patients with a high risk inherent to the condition, is recommended, no matter if there are already effective treatment options [11]. Therefore, we chose the study design with an active control group mainly for ethical reasons, which is in line with the decision framework from Gold et al. [11]. Generally, it can be discussed whether SHGs offer a cost-effective and widely available alternative to treat PNES patients. SHGs could be most valuable in rural areas where trained therapists are less available. These findings are also consistent with those of previous studies in other areas of behavioural health [21], pointing towards self-help approaches as a veritable alternative to therapist-delivered treatment [10].

There are several limitations to our study: The small sample size, mainly due to high drop out rates, led to insufficient power for small effects to reach statistical significance.

Nevertheless, our dropout rate is comparable to the dropout rates in previous studies with the same design and disease [7, 8]. However, we had a remarkably high dropout rate (study withdrawal) before the intervention groups started. From initially 67 screened patients, only 42 started the intervention in one of the two study arms. This high withdrawal rate can be partly explained by the non-blinded design, which triggered a 'nocebo’-effect in patients randomized in the control group arm. Six patients did not show up to the control group intervention because of the disappointment of being randomized to the 'wrong group'. For further studies, we recommend an improved communication, for example, neutral labelling of the study groups at recruitment, starting from a position of equipoise of the two treatment arms. This might help to reduce early withdrawal.

Previous studies have discussed the specific challenges of group psychotherapy interventions because the influence of the so-called 'proxies' (placebo effects evoked by family members or other patients who participate in psychotherapy groups) becomes even more virulent in group settings [23]. Of course, positive placebo effects are expected to rise when offering a group intervention, making our study design even more challenging. Further studies could change the study design by offering eHealth interventions, consisting of chatroom-based or blog-based group interventions with psychoeducational content. However, this might lower the effectiveness of the verum intervention because of weakened effects by proxies (e.g. other participants).

Regarding our CORDIS treatment program, some social aspects could improve the positive effect of the treatment compared with that in the active control group. We did not specifically include relatives in our treatment program, which could be a promising option due to the extreme involvement of the social environment of PNES patients [23].

In summary, CORDIS is a promising therapeutic tool for the treatment of PNES patients. In our German sample, the effects of CORDIS were most notable on the outcome of seizure severity 6 months after the treatment, compared with the effects of an active SHG. Our study widens the still small spectrum of clinical research projects regarding the treatment options for PNES patients. To further investigate the efficacy of our treatment program, a study with a higher statistical power (multicentric study) and an improved study design would be promising.
